# Intravenous and oral administration of the synthetic RNA drug, TY1, reverses heart failure with preserved ejection fraction in mice

**DOI:** 10.1007/s00395-024-01095-5

**Published:** 2024-12-31

**Authors:** Kazutaka Miyamoto, Xaviar M. Jones, Shukuro Yamaguchi, Alessandra Ciullo, Chang Li, Joshua Godoy Coto, Kara Tsi, Jessica Anderson, Ashley Morris, Eduardo Marbán, Ahmed Gamal-Eldin Ibrahim

**Affiliations:** Cedars-Sinai Medical Center, Smidt Heart Institute, 8700 Beverly Blvd, Los Angeles, CA 90048 USA

**Keywords:** Noncoding RNA, RNA therapeutics, Heart failure with preserved ejection fraction, Cell stress, Inflammation, Fibrosis, Hypertrophy

## Abstract

TY1, a synthetic non-coding RNA (ncRNA) bioinspired by small Y RNAs abundant in extracellular vesicles (EVs), decreases cGAS/STING activation in myocardial infarction and thereby attenuates inflammation. Motivated by the concept that heart failure with preserved ejection fraction (HFpEF) is a systemic inflammatory disease, we tested TY1 in a murine model of HFpEF. Intravenous TY1, packaged in a transfection reagent, reversed the cardiac and systemic manifestations of HFpEF in two-hit obese-hypertensive mice, without inducing weight loss. The effects of TY1 were specific, insofar as they were not reproduced by a control RNA of the same nucleotide content but in scrambled order. TY1 consistently suppressed myocardial stress-induced MAP kinase signaling, as well as downstream inflammatory, fibrotic, and hypertrophic gene pathways in heart tissue. TY1 not only prevented but actually reversed key pathological processes underlying HFpEF, with no evidence of toxicity. Most noteworthy from a practical perspective, the effects of intravenous TY1 were reproduced by feeding HFpEF mice an oral micellar formulation of TY1. As the prototype for a novel class of ncRNA drugs which target cell stress, TY1 exhibits exceptional disease-modifying bioactivity in HFpEF.

**Supplementary Information:**

The online version contains supplementary material available at 10.1007/s00395-024-01095-5.

## Introduction

Heart failure (HF) remains the greatest source of mortality and morbidity worldwide. Two distinct HF syndromes prevail: HF with reduced ejection fraction (HFrEF), which has many therapeutic options available, and HF with preserved ejection fraction (HFpEF), which remains refractory to many of the diverse medical interventions that ameliorate HFrEF [21]. Various lines of evidence indicate that HFpEF is not strictly a myocardial disorder [2, 16]. Obesity, a frequent comorbidity in HFpEF, induces inflammation which may contribute to HFpEF pathogenesis [3]. Consistent with this view, HFpEF patients improve when given anti-diabetic agents that induce weight loss [6]. We have recently developed a novel anti-inflammatory noncoding RNA drug, TY1, which exerts cardioprotection against myocardial infarction by attenuating innate immunity [10]. Not only is TY1 novel mechanistically, but also pharmacodynamically, being bioavailable either intravenously (IV) or orally [23]. Here we tested the therapeutic efficacy of TY1 in a two-hit obese-hypertensive murine model of HFpEF. Without inducing weight loss, TY1 reverses the key phenotypic hallmarks of HFpEF while coordinately suppressing the myocardial expression of inflammatory, fibrotic and hypertrophic genes. Equivalent benefits are seen with IV or oral TY1, facilitating the prospect of translation.

## Results

### TY1 exerts therapeutic bioactivity in a mouse model of HFpEF

To characterize the pharmacokinetics of TY1, we evaluated (by qPCR) the accumulation of TY1 in animals intravenously infused with TY1 formulated in a lipid transfection reagent (Fig. S1A). Administration of IV TY1 led to accumulation of TY1 in the heart, lungs, liver, kidney, spleen and plasma as early as 30 min post-infusion (Fig. S1B–F). TY1 abundance waned by 24 h in all tissues except the spleen, liver, and heart, which continued to retain TY1 levels measurable above background (Fig. S1B–F).

**Fig. 1:**
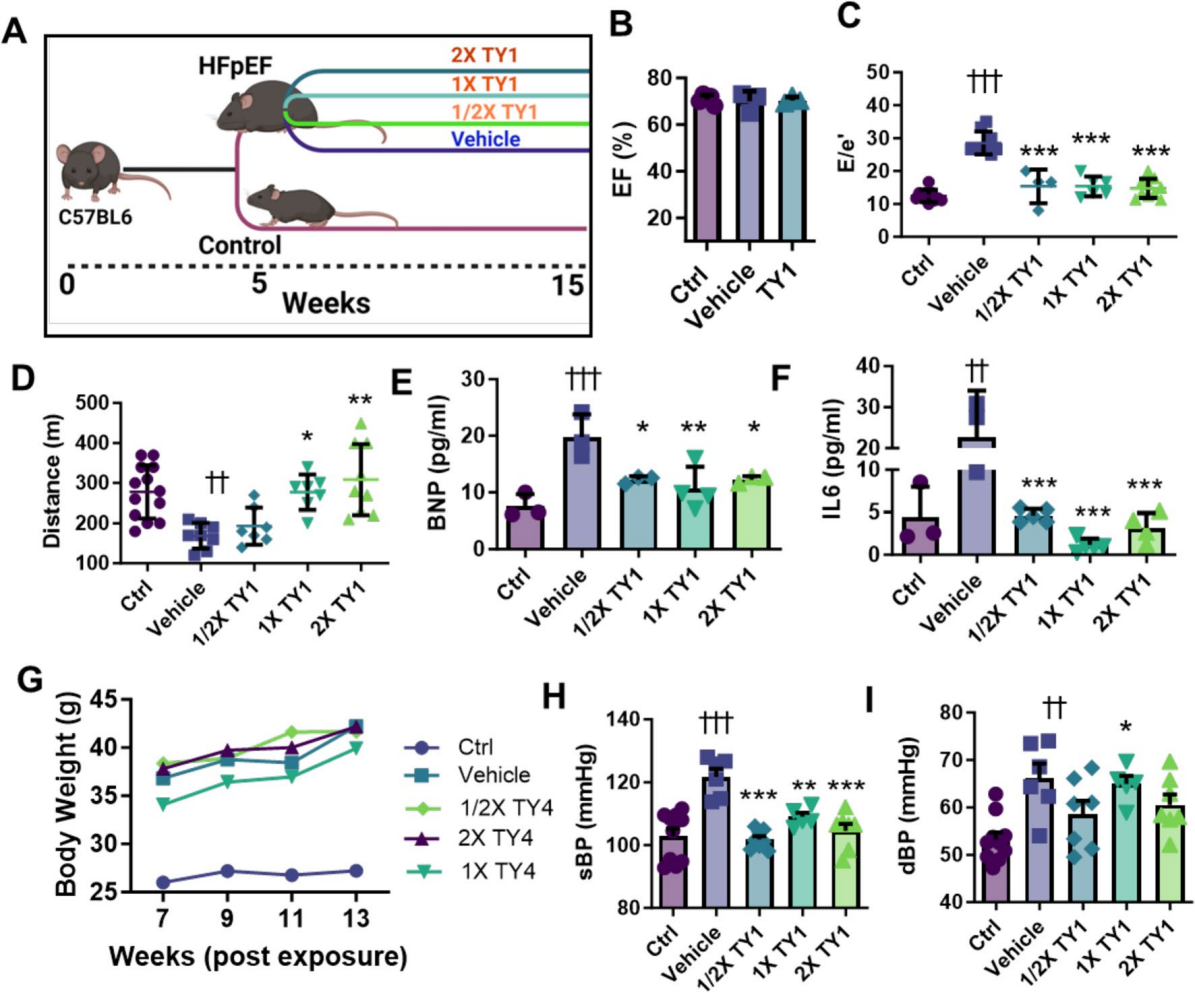
TY1 exerts therapeutic bioactivity in a cardiometabolic mouse model of HFpEF. **A**, Study outline for investigating the efficacy of intravenous administration TY1 in a mouse two-hit model of HFpEF. Wildtype c57BL/6 mice were placed on a high-fat diet and the nitric oxide synthase inhibitor Nω-Nitro-L-arginine methyl ester (L-NAME) for five weeks whereafter they were randomized to receive intravenous (retro-orbital) infusions of vehicle (saline; negative control) or TY1 (0.15 mg/kg) at frequencies of every other week, weekly, or biweekly (total of 10 weeks). **B**, confirmation of preserved ejection fraction in a representative sample of HFpEF mice from each group (n = 3 per group). TY1 administration reduced diastolic dysfunction as indicated by echocardiographic E/e´(n = 4–10 animals per group, **C**, improved exercise endurance (**D;** n = 7–10 per group) and reduced circulating brain natriuretic peptide (**E;** n = 3–4 per group) and IL6 levels (**F;** n = 3–4 animals per group). TY1 administration also exerted anti-hypertensive effects as shown by reductions in systolic, **H**, and diastolic pressure, **I** (n = 5–10 animals per group). Bars and lines represent group means and error bars represent s.d. Significance was determined by one-way ANOVA; *,^†^*P* < 0.05; **, ^††^*P* < 0.01; ***, ^†††^*P* < 0.001. *; denotes comparison between groups and vehicle, †; denotes comparison between groups and control

The cardiometabolic features of HFpEF are reproduced in a “two hit” mouse model induced by ingestion of a high-fat diet and the nitric oxide synthase inhibitor Nω-Nitro-L-arginine methyl ester (L-NAME) [17]. After 5 weeks, when signs of HFpEF are already evident [17], we administered TY1 at various dosing frequencies (bi-weekly, weekly, and twice weekly; 0.15 mg TY1/kg body weight per dose) and compared the findings to HFpEF animals that received only vehicle (saline; Fig. 1A). After 10 weeks of dosing, all groups had a normal ejection fraction (Fig. 1B), but TY1 exerted unique functional benefits, relative to vehicle, on two key manifestations of HFpEF: diastolic dysfunction (viz., E/e´ by echocardiography), which was attenuated in all groups receiving TY1 (Fig. 1C), and exercise endurance, which was normalized in animals receiving weekly or twice-weekly doses of TY1 (Fig. 1D). Meanwhile, circulating biomarkers of HF (brain natriuretic peptide [BNP]; Fig. 1E) and inflammation (IL6; Fig. 1F) were reduced in all groups receiving TY1. All these benefits occurred without changes in body weight, which was comparable in vehicle and all TY1 groups (Fig. 1G). TY1 did, however, exert an anti-hypertensive effect: systolic (Fig. 1H) and diastolic blood pressures (Fig. 1I) were reduced in groups receiving TY1 compared to vehicle. To determine if the anti-hypertensive effects of TY1 suffice to explain its benefits, we performed a separate group of experiments in which HFpEF mice were given the calcium channel blocker amlodipine (4 mg/kg/daily, oral; Fig. S2). With comparable reductions of blood pressure in the TY1 and amlodipine groups (Fig. S2A), circulating BNP levels were comparably attenuated (Fig. S2B). However, amlodipine differed from TY1 in its failure to lower circulating IL6 levels (Fig. S2C), attenuate diastolic dysfunction (Fig. S2D), or improve exercise endurance (Fig. S2E). These results, which echo the failure of amlodipine in HFpEF patients [7], reveal that TY1’s benefits are not entirely attributable to blood pressure control.

At face value, we interpret the effects of TY1 as being due to cellular uptake of an exogenous RNA with a specific sequence. Two additional experiments were performed to check the validity of these assumptions. First, we injected naked TY1 formulated without the transfection reagent DharmaFect®. In this instance, TY1 exerted no benefits on diastolic dysfunction (Fig. S3A), exercise endurance (Fig. S3B), or BNP levels (Fig. S3C). The lack of efficacy hints that naked TY1 may be degraded before it has a chance to act in vivo, but we have not explicitly tested this presumption. Second, to control for nonspecific RNA effects, we injected a synthetic RNA with the same nucleotide content as TY1 but in randomized order [10]. Such a scrambled RNA (Scr), formulated with the transfection reagent DharmaFect®, had no therapeutic benefit (as demonstrated here by the lack of an effect on diastolic dysfunction; Fig. S3D). Previous toxicity studies demonstrated that neither TY1 nor Scr exhibited any evidence of toxicity in healthy animals [10, 23]. Here, mice that received Scr vs. vehicle behaved comparably in terms of heart function and exercise tolerance, while TY1 actually improved those indices of health. Thus, these new chemical entities appear not to elicit the generalized immune reactions that have plagued a number of other ncRNA therapeutic candidates [20]. Taken together, the findings indicate that TY1 is highly effective in reversing various key phenotypic manifestations of HFpEF. With IV infusion, both a lipid coat and a specific sequence are required for therapeutic efficacy.

### Orally-administered TY1 recapitulates the benefits of intravenous TY1 in HFpEF

**Fig. 2:**
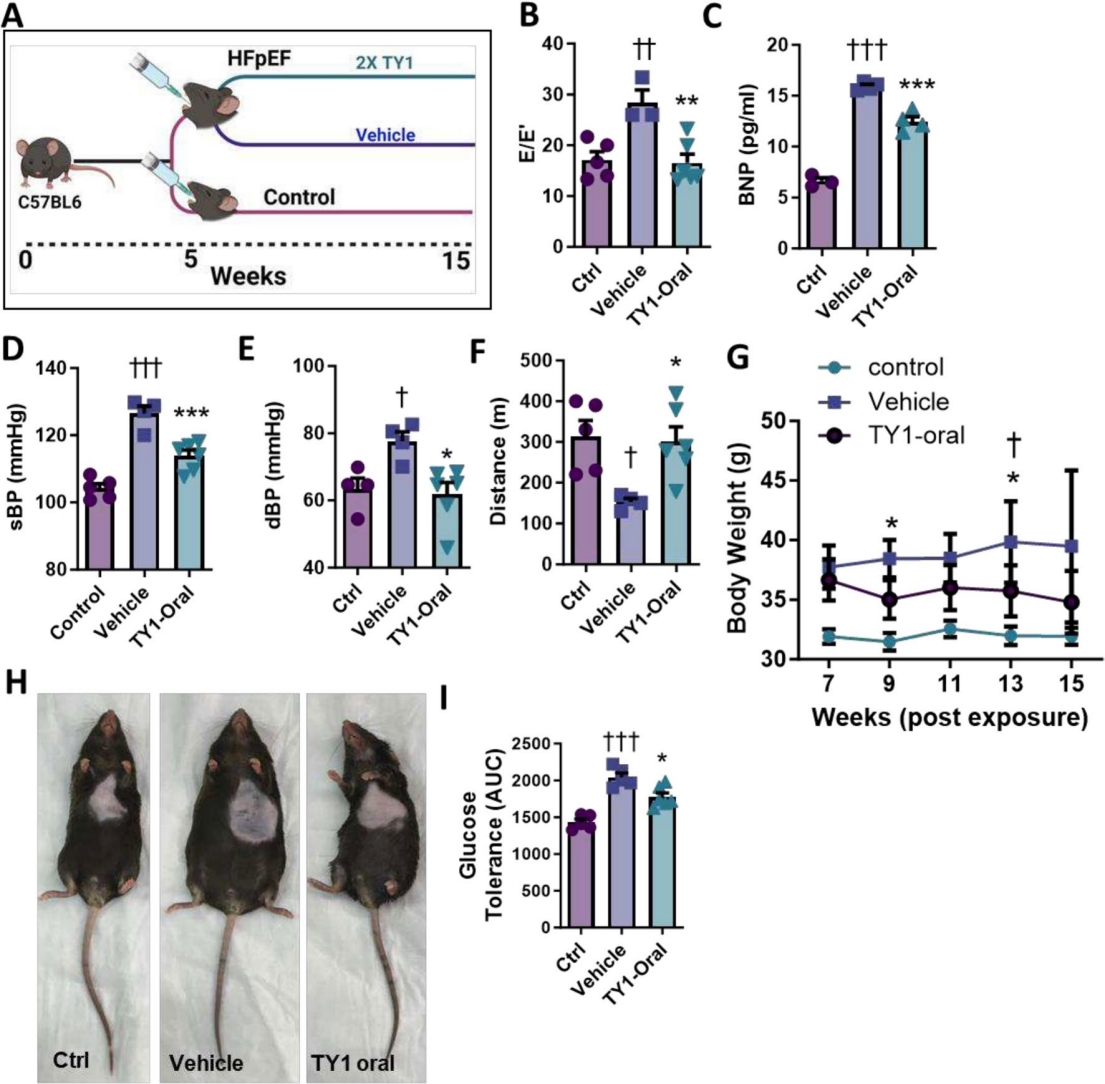
Oral Administration of TY1 recapitulates the benefits of Intravenous TY1 in HFpEF. **A**, Study outline for investigating the efficacy of oral administration of TY1 in the mouse two-hit model of HFpEF. **B**, animals receiving orally-formulated TY1 had preserved diastolic function, **B**, reduced circulating BNP levels, **C**, systolic and diastolic blood pressure, **D**, **E**, and preserved exercise endurance, **F** compared to HFpEF mice receiving vehicle. HFpEF mice fed TY1 also showed marginal weight loss, **G**, **H**, and reduced glucose tolerance compared to vehicle-fed animals, **I**. Bars and points represent group means and error bars represent s.d. Significance was determined by one-way ANOVA; *,^†^*P* < 0.05; **, ^††^*P* < 0.01; ***, ^†††^*P* < 0.001. *; denotes comparison between groups and vehicle, †; denotes comparison between groups and control

Encapsulation of TY1-DharmaFect® into casein-chitosan micelles yielded a novel formulation that, when given orally to rodents, recapitulates the cardioprotective effects of IV TY1 [23]. An orally effective formulation would be particularly advantageous in the translation of TY1 to human HFpEF, given the chronic, epidemic nature of this disease. To investigate the efficacy of orally-formulated TY1 (TY1-oral), we fed animals TY1-oral twice weekly (cf. Figure 1) for ten weeks (Fig. 2A). Similar to the findings with IV TY1 administration, HFpEF animals fed TY1-oral showed attenuation of diastolic dysfunction (Fig. 2B) and hypertension (Fig. 2C, D), reduced circulating BNP levels (Fig. 2E), and normalized exercise endurance (Fig. 2F). Unlike IV TY1, however, animals fed TY1-oral showed modest weight loss (Fig. 2G, pooled data; Fig. 2H, representative images). Animals fed TY1-C2 also had improved glucose tolerance compared to vehicle (Fig. 2I). Taken together, these findings extend previous observations [23] in showing that orally formulated TY1 exerts therapeutic benefits comparable to those of IV administration, but this time, in a chronic disease model, unlike the prior single-dose administration in acute myocardial infarction.

### TY1 targets stress-induced MAPK signaling to attenuate tissue damage in HFpEF

**Fig. 3:**
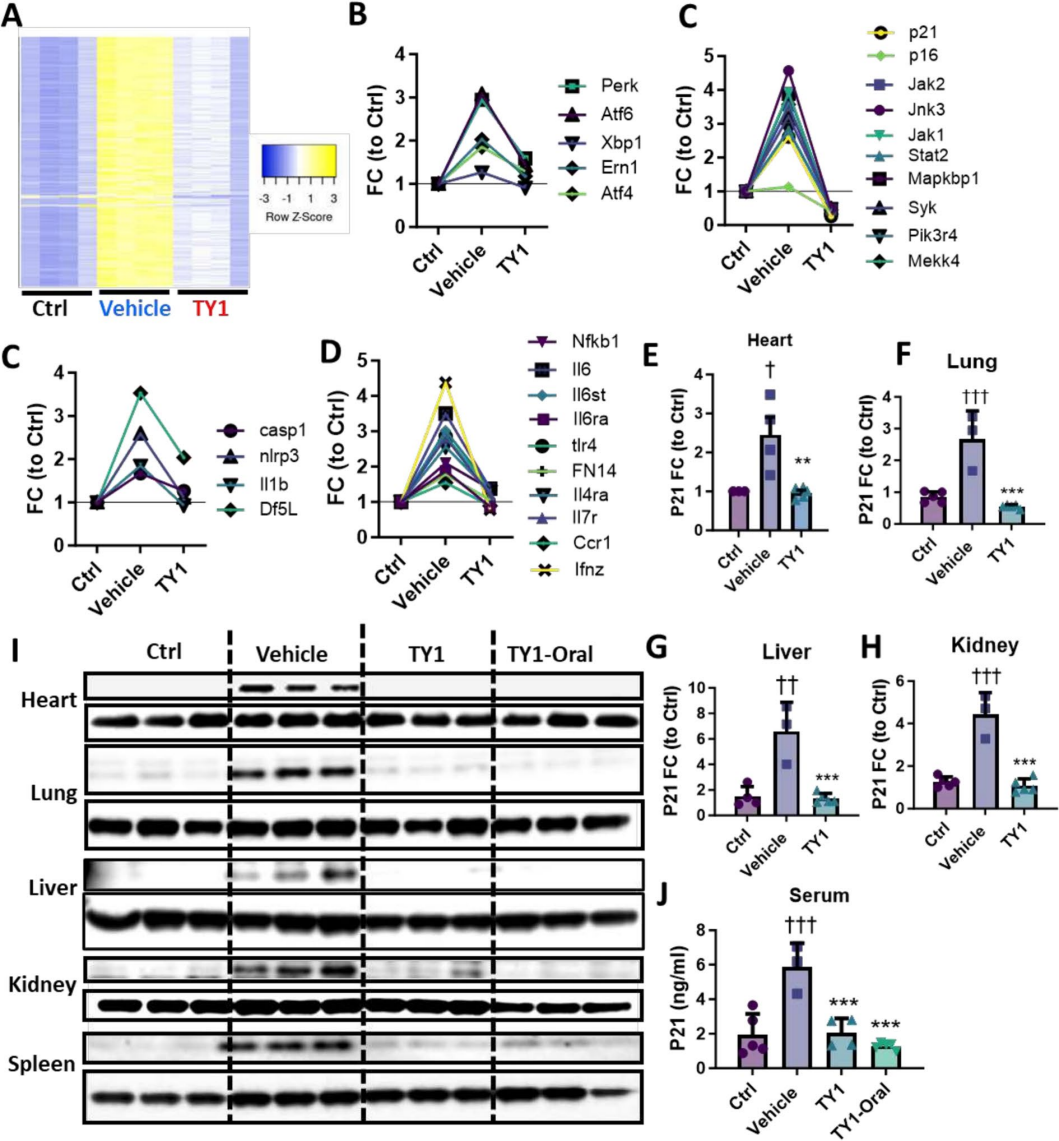
TY1 targets stress-induced MAPK signaling to attenuate tissue damage. **A,** Heatmap of RNA sequencing data from control (Ctrl; normal hearts) and HFpEF mouse hearts, the latter comparing vehicle vs TY1 infusions. TY1 suppresses cell stress signaling in HFpEF hearts (compared to vehicle) back to nearly Ctrl levels, starting with endoplasmic reticulum (ER) stress luminal receptors, **B**, stress-induced MAP kinase signaling, **C**, inflammasome signaling, **D,** and downstream inflammatory markers, **E** (n = 5 animals per group). P21 is suppressed in TY1-treated HFpEF mice as shown by gene expression, **F**-**J**, and protein in tissue, **K,** and serum, **L**, compared to the control (n = 3 hearts per group). Bars and points represent group means and error bars represent s.d. Significance was determined by one-way ANOVA; *,^†^*P* < 0.05; **, ^††^*P* < 0.01; ***, ^†††^*P* < 0.001. *; denotes comparison between groups and vehicle, †; denotes comparison between groups and control

**Fig. 4:**
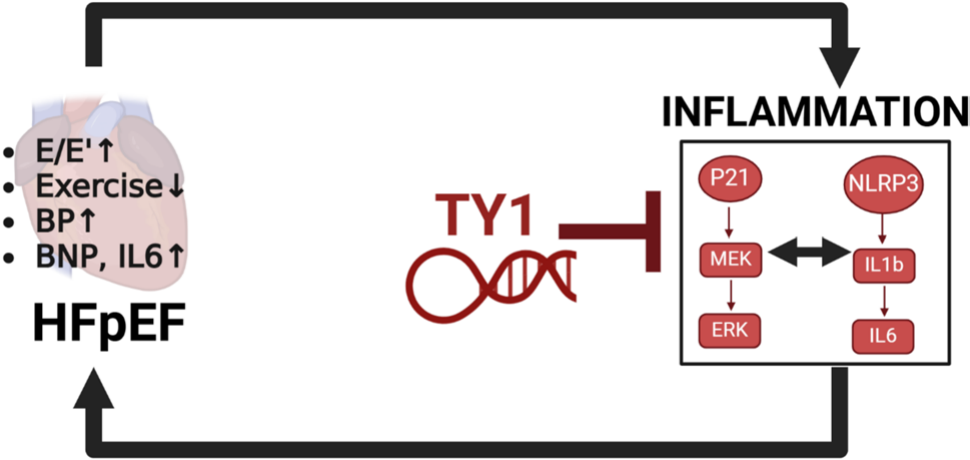
Inflammatory pathways drive the development of cardiometabolic manifestations that sustain, and perhaps precipitate, heart failure with preserved ejection fraction (HFpEF), as modeled in mice fed a high-fat diet and L-NAME. Novel noncoding RNA drug TY1 reverses the key phenotypic abnormalities of HFpEF in such mice. TY1 works when administered either intravenously or orally, without inducing major weight loss

HFpEF broadly alters the myocardial transcriptome[8, 13]. We find that TY1, in a highly coordinated manner, resets myocardial gene expression towards normal levels in two-hit mice (heat map, Fig. 3A). The coordinated changes included normalization of gene families relevant to structure (Fig. S4A), canonical Wnt signaling (Fig. S4B), and ion transport (Fig. S4C-E). The most upstream changes involved endoplasmic reticulum stress, in particular intraluminal stress sensors relevant to the unfolded protein response (Fig. 3B). Downstream regulators, including stress-induced MAP kinase signaling (Fig. 3C), inflammasome (Fig. 3D) and inflammatory mediators (Fig. 3E), were also normalized. A marker of stress-induced MAPK signaling and senescence, p21, was markedly increased in HFpEF hearts but suppressed by in vivo TY1 therapy at both the transcript (3F-H, in tissue) and protein levels (in both tissue [Fig. 3I] and serum [Fig. 3J]). Indeed, pathologic MAPK signaling has been previously implicated in myocyte stress, hypertrophy, and apoptosis [15]. These findings are entirely consistent with the hypothesis that TY1 is beneficial in HFpEF by virtue of its ability to blunt inflammation at a very basic level [10] (Fig. 4).

## Discussion

TY1 is the lead compound for a new class of drugs targeting cell stress pathways, and exhibiting exceptional disease-modifying bioactivity. Here we demonstrate that TY1 is remarkably effective against HFpEF, a disease marked by inflammation, fibrosis, and hypertrophy [22]. The benefits of TY1 are specific: a scrambled version had no effect, and formulating the IV drug without a transfection reagent neutralized the therapeutic benefits. The only drug classes shown to be effective in HFpEF to date are the SGLT2 inhibitors and GLP1 agonists, and even they are limited in efficacy, insofar as mortality is not decreased, at least not in the trials reported to date [1, 4, 5, 12, 19]. Here we have shown that a novel, biologically-based drug is effective in a cardiometabolic rodent model of HFpEF, motivating further development of TY1 as a therapeutic candidate for this complex multisystem illness. Future studies will further dissect the broader potential effects of TY1 on HFpEF including, perhaps, improvement of coronary artery blood flow, which has been implicated as a driver of heart failure progression [9]. The focus here has been on HFpEF, but the same cell stress pathways figure prominently in diverse pathologies. Thus, TY1 is arguably worth testing in models of other diseases driven by inflammation, fibrosis, and/or hypertrophy, including, but not limited to, scleroderma and Duchenne muscular dystrophy.

TY1 is the product of a novel discovery paradigm: mining EVs for ncRNAs previously unrecognized as bioactive, then using those natural compounds as bioinspiration for new chemical entities [14]. Unlike previous ncRNA drugs, which were designed with a specific genetic target in mind [18], TY1 was created from a natural product of unclear mechanism. We thus worked backwards, first documenting disease-modifying bioactivity and then probing the mechanism. In contrast with the existing RNA pharmacopeia, TY1 does not work as a small interfering RNA, an aptamer or an antisense oligonucleotide, making it the prototype for a new class of RNA drugs. Recent findings reveal that TY1 upregulates the expression of TREX1, a central control point for cytosolic DNA and, hence, genotoxic stress in tissue [10]. Such a fundamental effect helps rationalize the broad, coordinated effects of TY1 on gene expression in HFpEF heart tissue which we describe here. While our findings are consistent with TY1-induced TREX1 upregulation and attenuation of cGAS/STING signaling, we have not yet probed the role of this previously-unsuspected innate immunity pathway in HFpEF. Regardless, our findings underscore the potential of investigating otherwise obscure, EV-derived ncRNA molecules in developing next-generation therapeutic candidates.

## Methods

### Experimental animals

All studies were performed at Cedars-Sinai Medical Center in accordance with the Institutional Animal Care and Use Committee guidelines.

### Intravenous TY1 pharmacokinetics

In vivo, experimental protocols were performed on 10- to 12-week-old male C57BL76J mice (Jackson Laboratory). Mice were housed under pathogen-free conditions in a temperature-controlled room with a 12-h photoperiod. TY1 (0.15 mg/kg) or PBS in Dharmafect (Horizon) was resuspended in 50 μl of saline and injected into the retro-bulbar space of healthy mice anesthetized by isofluorane. At multiple time-points (15 min, 30 min, 60 min, 3 h, 24 h, 48 h) mice were sacrificed and tissues (heart, lung, liver, kidney, spleen, thymus, white adipose tissue, soleus muscle, tibialis anterior muscle, Peyer’s patches, inguinal lymph nodes, axillary lymph nodes, mesenteric lymph nodes, brain) were removed (without prior perfusion), washed in PBS, and flash frozen in liquid nitrogen until further processing was performed. Femurs and tibias were isolated and flushed with PBS to collect cells in the bone marrow. Cells were filtered with 70 µm cell strainer (431,751, Corning), treated with ACK Lysing Buffer (A1049201, ThermoFisher Scientific) for 30 s, neutralized with excess PBS, centrifuged 500 g 10 min at 4 °C, then resuspended in PBS and frozen at -80 °C until further processing was performed. Blood was collected from the heart (using K2 EDTA tubes) and PBMCs and plasma were isolated using SepMate PBMC Isolation Tubes (85,415, StemCell Technologies) and Ficoll-Paque PLUS density gradient media (17,144,002, Cytiva) according to SepMate manufacturer’s protocol. Plasma was frozen at -80 °C and PBMCs were used for fractionation or resuspended in Qiazol (Qiagen) and frozen at -80 °C until further processing was performed.

#### CD3ε and CD11B fractionation

PBMCs were resuspended in MACS buffer from a 1:20 dilution of MACS BSA Stock Solution (130–091-376, Miltenyi Biotec) in autoMACS Rinsing Solution (130-091-222, Miltenyi Biotec) according to manufacturer’s protocol. CD3ε cells were isolated from PBMCs using CD3ε MicroBead Kit (130-094-973, Miltenyi Biotec) with the OctoMACS Separator (130-042-109, Miltenyi Biotec) and LD Columns (130-042-901, Miltenyi Biotec) according to manufacturer’s protocol. CD11B cells were isolated from the CD3ε- fraction using CD11B MicroBeads UltraPure (130-126-725, Miltenyi Biotec) with the OctoMACS Separator and MS Columns (130-042-201, Miltenyi Biotec) according to manufacturer’s protocol. CD3ε + fraction, CD11B + fraction, and double negative fraction were resuspended in Qiazol and frozen at -80 °C until further processing was performed.

#### Tissue RNA isolation and TY1 qPCR assay

Tissues were homogenized in Qiazol using Bead Ruptor 12 (OMNI 194 International) with RNase free steel beads. Total RNA was extracted from mouse cells and tissue or plasma using miRNeasy mini kit (217,004, QIAGEN) or miRNeasy Serum/Plasma kit (217,184, QIAGEN) respectively according to the manufacturer’s protocol. RNA was resuspended in 35 µl RNase free water for the organs and bone marrow cells, 25 µl RNase free water for PBMCs or fractionated cells, and 20 µl RNase free water for plasma samples; RNA concentration and purity were determined using a NanoDrop Spectrophotometer (Thermo Scientific). cDNA was synthesized from RNA using miRCURY LNA RT Kit (339,340, QIAGEN) using UniSp6 RNA spike-in in each reaction, according to the manufacturer’s protocol. cDNA was diluted 1:60 and Real-time PCR was performed in duplicate using the following kits and primers: miRCURY SYBR Green PCR Kit (339,345, QIAGEN), miRCURY LNA miRNA PCR Assay (339,306, QIAGEN), miRCURY LNA miRNA Custom PCR Assay (339,317, QIAGEN). qPCR was done on QuantStudio 12 K Flex or QuantStudio 6 Flex system (Applied Biosystems) using the following protocol: initial activation step 2 min 95 °C, 2-step cycling (Denaturation 10 s 95 °C, Annealing 1 min 56 °C) for 40 cycles, then melt curve formation (Melting 15 s 95 °C, Annealing 1 min 60 °C, Deactivation 15 s 95 °C). Analysis was performed by calculating fold change as 2^(−ΔΔCT)^ for TY1, normalizing TY1 values to UniSp6 values. Analysis for plasma was performed by normalizing TY1 values to U6 values. Results are presented in Log_2_FC.

### Mouse two-hit model of heart failure with preserved ejection fraction

Eight to ten-week-old male C57BL/6 mice were obtained from Charles River laboratories. Mice were housed under controlled with a 12:12-h light–dark cycle and had unrestricted access to food (2916, Teklad for Control groups and D12492, Research Diet for High Fat Diet groups) and water. L-NAME (Nω-Nitro-L-arginine methyl ester hydrochloride, N5751, Millipore sigma 0.5 g/l) was added to drinking water after adjusting the pH to 7.4.

### TY1 synthesis and quality control

Research-grade TY1 is synthesized commercially by Integrated DNA Technologies (IDT, Inc.) using a proprietary solid-state synthesis method followed by standard desalting. IDT performs quality control on all custom-manufactured RNA products using electrospray-ionization mass spectrometry, optical density (OD_260_), and melting temperature. Below is a table of taken from the specification sheets of various batches of synthesized RNA TY1 oligos.

**Table:** 

Order ID	Extinction coefficient (L/mole•cm)	OD_260_	T_m_ (°C)	nmoles	mg
17,818,277	240,800	18.7	63.0	77.6	0.61
17,009,804	241,500	19.1	63.0	79.3	0.62
16,961,714	246,100	18.4	63.2	75	0.58

For transfection and IV formulation, the RNA oligo is mixed with Dharmafect (Perkin Elmer), a proprietary cationic lipid for the transfection of small RNAs. For oral formulation, the TY1-Dharmafect complex is further encased in a casein-chitosan micelle. Chitosan, casein, and acetic acid are all sourced from Sigma Aldrich at research grade.

### Formulations of TY1 for intravenous and oral delivery

As described, TY1 was formulated using lipid transfection by admixture with DharmaFect® per manufacturer’s instructions (Perkin Elmer). This formulation sufficed for IV use (0.15 mg/Kg, retroorbitally once every two weeks, once a week, or twice a week). In some experiments as indicated, DharmaFect® was omitted to determine if it was required for IV efficacy. For oral administration, as described previously, TY1-DharmaFect® (at a dose of 0.2 mg/kg) is mixed with a casein solution. Micelles are formed by the addition of chitosan solution under acidic conditions. The suspension was incubated at room temperature one hour prior to administration by oral gavage (given twice weekly).

#### Echocardiography

Cardiac function and morphology were assessed under general anesthesia by transthoracic echocardiography using Vevo 3100 (VisualSonics). Apical four-chamber views were performed for diastolic function measurements using pulsed-wave and tissue Doppler imaging at the level of the mitral valve. During echocardiography, body temperature of mice was controlled, and isoflurane was reduced to under 1.0% and adjusted to maintain a heart rate in the range of 420–470 bpm.

### Western blot

Protein extracts from mouse tissue were prepared by lysis in RIPA buffer (89,900, Thermo Scientific) containing protease and phosphatase inhibitor (78,442, Thermo Scientific). Protein samples (normalized value between 10 and 30 μg) were separated for gel electrophoresis (NUPAGE 4%-12% Bis–Tris gel, NP0336 Thermo Fisher Scientific) and transferred to nitrocellulose membranes using Trans-Blot Turbo Transfer System, Bio-Rad. Proteins were detected with the following primary antibodies: p21 (ab109199, abcam) and GAPDH (3683S, Cell Signaling technology).

### RNA isolation and quantitative PCR

Total RNA was extracted from mouse tissue using RNeasy plus kit (74,136, QIAGEN) and Maxtract High density (129,056, QIAGEN). cDNA was synthesized from RNA using High capacity cDNA reverse transcription kit (4,368,813, Applied Biosystems) according to the manufacturer’s protocol. Real-time PCR (QuantStudio 12 K Flex Real-Time PCR system; Thermo Fisher Scientific) was performed in triplicate using the following TaqMan Gene Expression Assay probes (Cat# 4,331,182; with corresponding Assay IDs);

**Table:** 

Species	Gene	Assay ID
Mouse	P21	Mm04205640_g1

Differential gene expression analysis was done using the ddCt method.

### RNA sequencing

Cell and tissue RNA samples were sequenced at the Cedars**-**Sinai Genomics Core as described previously[11]. Total RNA was analyzed using an Illumina NextSeq 500 platform.

#### Library preparation and sequencing

Total RNA samples were assessed for concentration using a Qubit fluorometer (ThermoFisher Scientific, Waltham, MA) and for quality using the 2100 Bioanalyzer (Agilent Technologies, Santa Clara, CA). Library construction was performed using the QIASeq Stranded RNA Library kit (Qiagen, Hilden, Germany) with QIAseq FastSelect—rRNA HMR Kit (Qiagen) for ribosomal RNA depletion. Library concentration was measured with a Qubit fluorometer and library size on a Bioanalyzer. Libraries were multiplexed and sequenced on a NovaSeq 6000 (Illumina, San Diego, CA) using 75 bp single-end sequencing. On average, approximately 50 million reads were generated from each sample.

#### Data analysis

Raw sequencing data were demultiplexed and converted to fastq format by using bcl2fastq v2.20 (Illumina, San Diego, California). Then reads were aligned to the GRCm38 reference genome (http://www.gencodegenes.org) using STAR (version 2.6.1)4 with default parameters. Gene expression was quantified by RSEM (version 1.2.28)5 to generate a raw count expression matrix with gene identities as rows and samples as columns. DESeq2 (version 1.26.0)6 was used to normalize the raw count expression and correct the batch effect.

### Statistical analysis

Statistical parameters including the number of samples (n), descriptive statistics (mean and standard deviation), and significance are reported in the figures and figure legends. Differences between groups were examined for statistical significance using the Student’s t-tests or analysis of variance with Tukey’s post hoc test. Differences with *p* values < 0.05 were regarded as significant.

## Supplementary Information

Below is the link to the electronic supplementary material.

Supplementary file 1 (DOCX 426 KB)

## Data Availability

Data is available upon request.
