# Supplementary material for: Intravenous and oral administration of the synthetic RNA drug, TY1, reverses heart failure with preserved ejection fraction in mice
Source: Basic Res Cardiol. 2024 Dec 31;120(2):363–71. doi: 10.1007/s00395-024-01095-5 (PMC11976778; doi:10.1007/s00395-024-01095-5)
Supplement: Supplementary file 1 — (DOCX 426 KB) [file 395_2024_1095_MOESM1_ESM.docx]

**
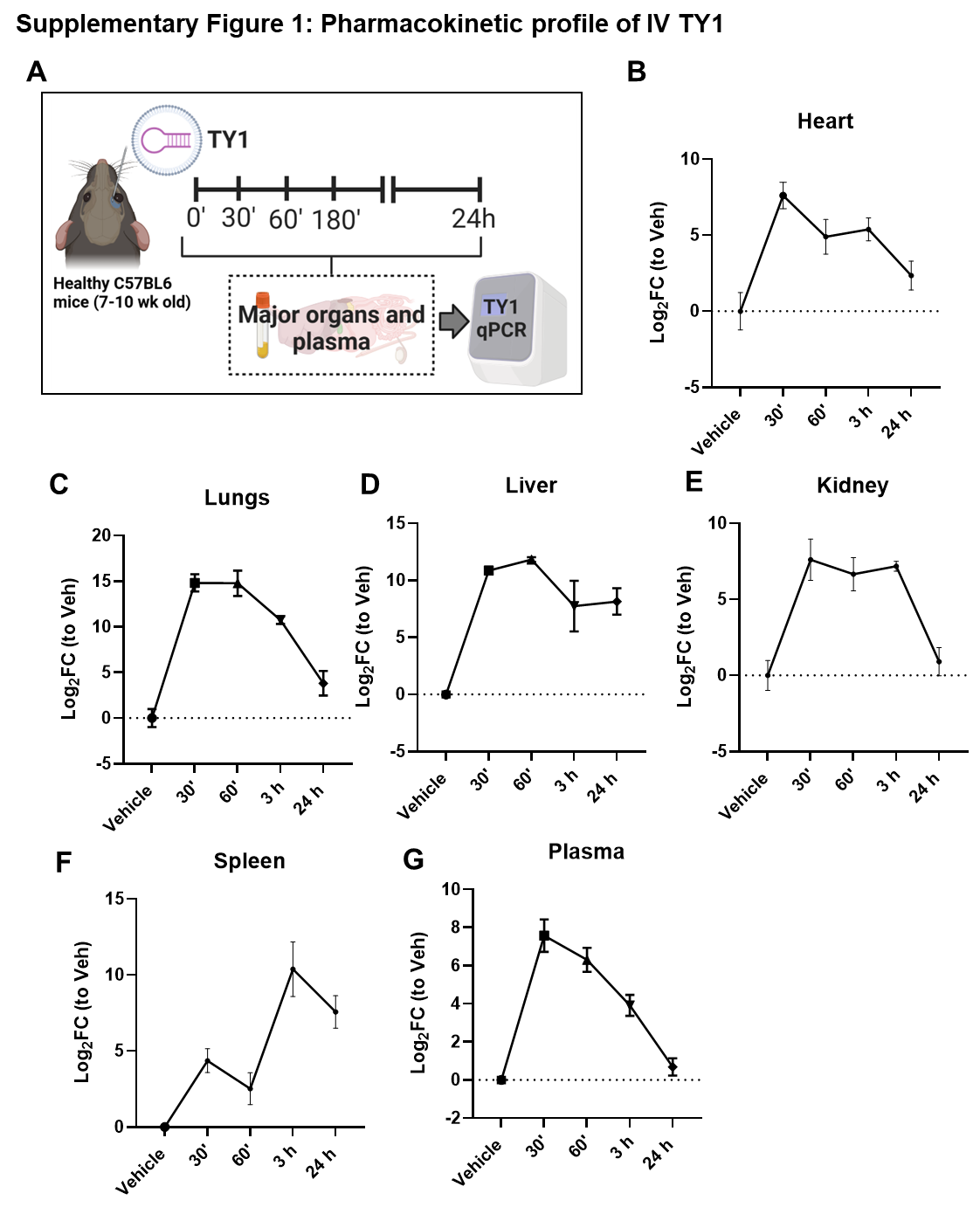
Supplementary Figures**

**
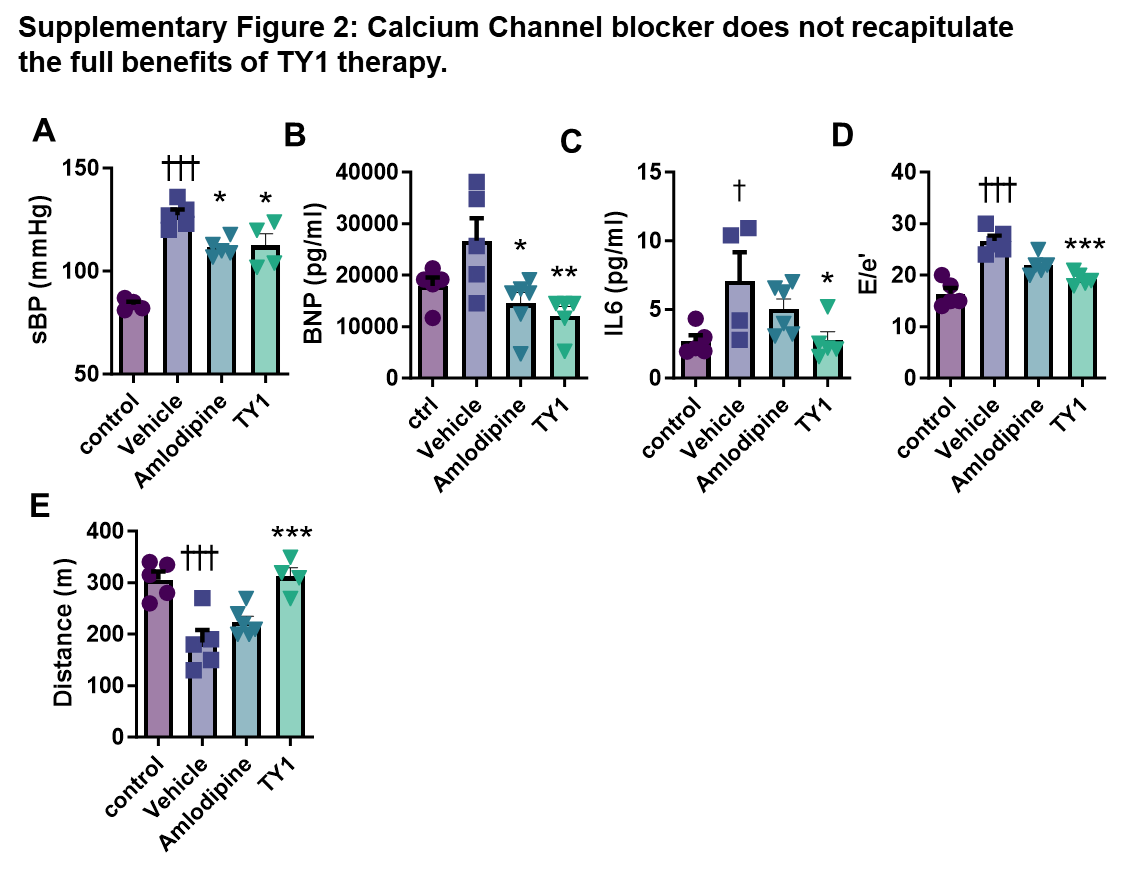

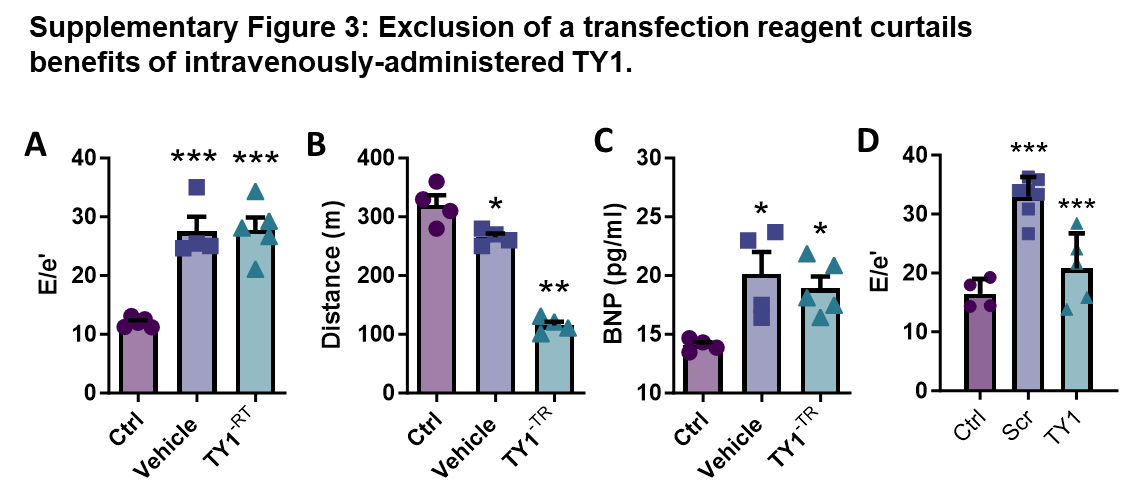

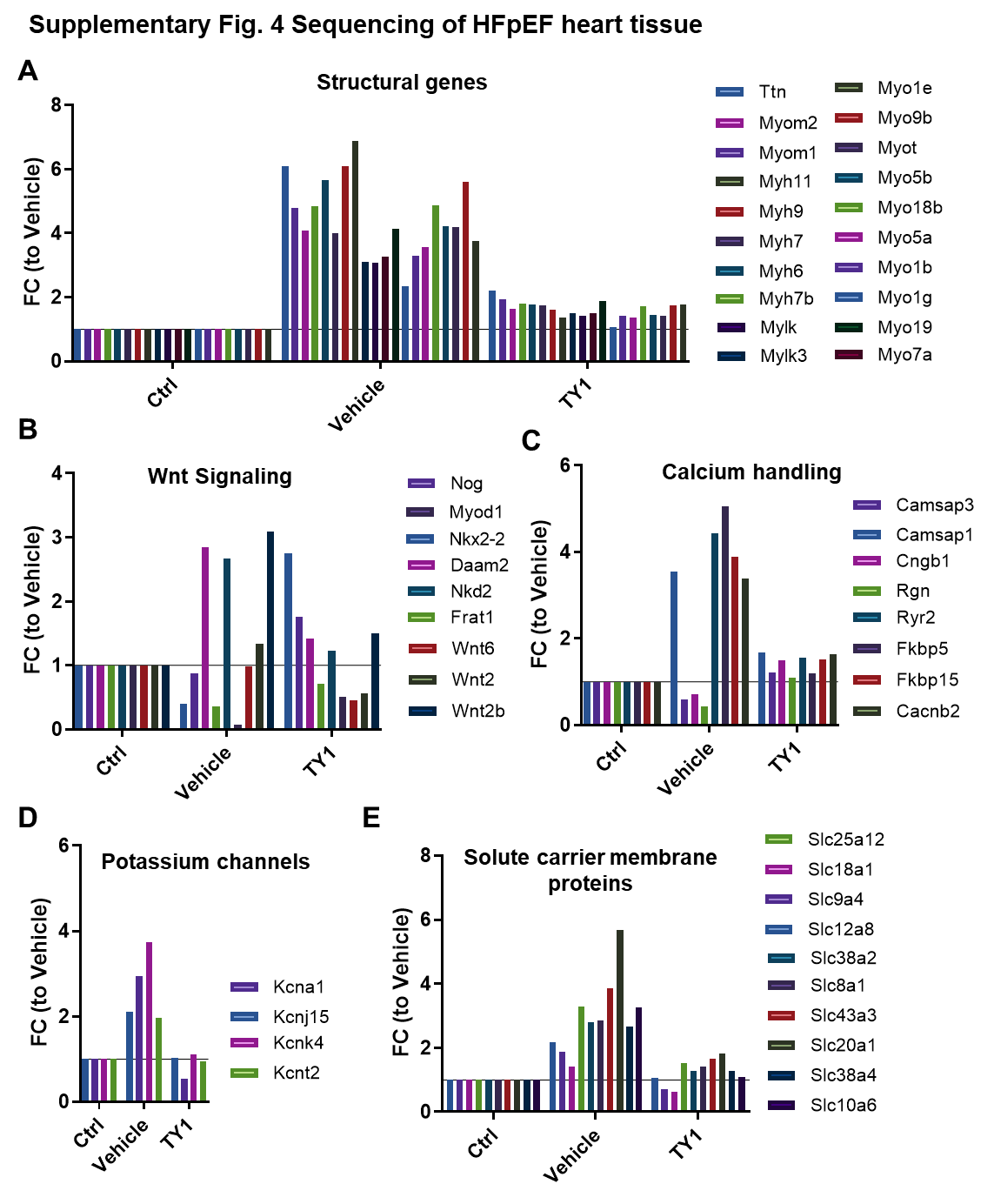
**

**Supplementary Fig. 1: Pharmacokinetic profile of IV TY1 in healthy mice**

**A**, Schematic of IV TY1 pharmacokinetic study. Healthy C57BL6 mice were given a single IV infusion of TY1 and animals (n=4-5 animals per timepoint per group) were sacrificed at various time points to collect heart, lung, liver, kidney, spleen and plasma to assess TY1 abundance by qPCR (as compared to a vehicle control). **B**-**G**, abundance of TY1 in various tissue at multiple time points expressed as log2 fold change compared to vehicle animals. Points reperesent group means and error bars represent SEM.

**Supplementary Fig. 2: Therapeutic effects of TY1 in HFpEF are not entirely attributable to anti-hypertensive effects. A**, **B**, HFpeF mice receiving daily oral administration of the calcium channel blocker amlodipine (4 mg/kg) had anti-hypertensive effects comparable to mice receiving twice-weekly intravenous infusions of TY1. However, unlike the amlodipine group, animals given TY1 has significantly less circulating BNP and IL6 (**B** and **C**, respectively), diastolic dysfunction (though a trend towards lower E/e’ was evident in the amlodipine group. **D**), and exercise endurance, E. N=4-5 animals per group. Bars represent group means and error bards represent s.d. *,^†^P<0.05; **, ^††^P<0.01; ***, ^†††^P<0.001. *; denotes comparison between groups and vehicle, †; denotes comparison between groups and control.

**Supplementary Fig. 3 Salutary effects of TY1 in HFpEF is not due non-specific effects**

Loss of TY1 therapeutic bioactivity on diastolic dysfunction in HFpEF animals receiving TY1 without transfection reagent (-TR) as shown by exercise endurance, **A**, diastolic dysfunction, **B**, and circulating BNP levels, **C**. Lack of therapeutic bioactivity of a scramble sequence compared to TY1 in HFpEF animals as shown by lack of improvement in diastolic dysfunction in HFpEF animals (n=5 animals per group). **D**, Significance was determined by one-way ANOVA; *,^†^P<0.05; **, ^††^P<0.01; ***, ^†††^P<0.001. *; denotes comparison between groups and vehicle, †; denotes comparison between groups and control.

**Supplementary Fig. 4 Sequencing of HFpEF heart tissue** Sequencing results showing TY1-induced normalization of HFpEF-dysregulated genes (compared to vehicle) including structural, (**A**), developmental (**B**), calcium handling (**C**), potassium channels (**D**), and solute carrier membrane proteins (**E**) (n=5 hearts per group).
